# Two Novel Mutations and a *de novo* Mutation in *PSEN1* in Early-onset Alzheimer’s Disease

**DOI:** 10.14336/AD.2018.1109

**Published:** 2019-08-01

**Authors:** Yu-Sheng Li, Zhi-Hua Yang, Yao Zhang, Jing Yang, Dan-Dan Shang, Shu-Yu Zhang, Jun Wu, Yan Ji, Lu Zhao, Chang-He Shi, Yu-Ming Xu

**Affiliations:** ^1^Department of Neurology, The First Affiliated Hospital of Zhengzhou University, Zhengzhou University, Zhengzhou, Henan, China.; ^2^Department of Neurology, Luoyang Central Hospital affiliated to Zhengzhou University, Henan, China

**Keywords:** Early-onset Alzheimer’s disease, EOAD, Mutation, *PSEN1*

## Abstract

Presenilin 1 (*PSEN1*), presenilin 2 (*PSEN2*), and amyloid precursor protein (*APP*) mutations are responsible for autosomal dominant early-onset Alzheimer’s disease (AD-EOAD). To analyze the phenotypes and genotypes of EOAD patients, we performed comprehensive clinical assessments as well as mutation screening of *PSEN1*, *PSEN2*, and exons 16 and 17 of *APP* by Sanger sequencing in the three Chinese EOAD families. We identified two novel mutations of *PSEN1* (Y256N and H214R) in samples from these families, and a *de novo* mutation of *PSEN1* (G206V) in a patient with very early-onset sporadic Alzheimer’s disease. A combination of bioinformatics tools based on evolutionary, structural and computational methods predicted that the mutations were all deleterious. These findings suggest that *PSEN1* Y256N, H214R, and G206V need to be considered as potential causative mutations in EOAD patients. Further functional studies are needed to evaluate the roles of these mutations in the pathogenesis of AD.

Alzheimer’s disease (AD) is the most common form of dementia and has a high incidence rate among the elderly people [1]. AD can be categorized into early-onset Alzheimer’s disease (EOAD, <65 years of age) and late-onset AD (LOAD, >65 years of age). Most AD cases are LOAD, whereas EOAD is reported quite rarely, accounting for 5-10 % of all the AD cases [2]. To date, three genes responsible for autosomal-dominant EOAD (AD-EOAD) have been identified: presenilin 1 (*PSEN1*), presenilin 2 (*PSEN2*), and amyloid precursor protein (*APP*) [3, 4]. Besides, there are also several *de novo* EOAD mutations detected in very early-onset sporadic cases of AD [5-10].

Mutations in *PSEN1* are the most frequent mutations in AD-EOAD patients. The *PSEN1* gene (NM_000021.3) contains 10 coding exons and three noncoding exons. It encodes the PSEN1 protein, which consists of nine transmembrane (TM) domains and a hydrophilic loop (HL) region [11]. PSEN1 is the major component of the catalytic subunit of the γ-secretase complex performing APP cleavage [12]. Mutations in *PSEN1* could lead to over-production of amyloid β 1-42 and amyloid deposits in the brain [13]. Until now, more than 200 pathogenic mutations in the *PSEN1* gene have been reported. EOAD cases with *PSEN1* mutations usually occur at 40-60 years of age, but several *PSEN1* mutations have also been reported to be associated with very early-onset AD (vEOAD; <35 years of age) [14]. Nonetheless, there are still mutations that remain unidentified along with the rarely reported *de novo* mutations [15]. In this study, we aimed to present the phenotypic and genetic analysis of three Chinese EOAD families.

## MATERIALS AND METHODS

### Subjects

Three Chinese EOAD families were enrolled in the study. EOAD was diagnosed independently by two neurologists on the basis of the criteria of the National Institute of Neurological and Communicative Disorders, and the Stroke-Alzheimer’s Disease and Related Disorder Association (NINCDS-ADRDA) [16]. Each patient was assessed via a series of neuropsychological scales, including the Mini-Mental State Examination (MMSE), Montreal Cognitive Assessment (MoCA), Clinical Dementia Rating (CDR), and Alzheimer’s Disease Assessment Scale-cognitive (ADAS-cog). Besides, 200 healthy Chinese volunteers were enrolled as controls. The study protocol was approved by the Ethics Committee of the First Affiliated Hospital of Zhengzhou University. Written informed consent was obtained from each participant.

### Genetic analysis

Genomic DNA was extracted from the peripheral blood leukocytes. After PCR amplification of relevant DNA fragments from purified genomic DNA samples, the coding region and exon-intron boundaries of* PSEN1*, *PSEN2* and *APP* (exons 16, 17) were subjected to Sanger sequencing. Alignment and analysis were carried out using the DNAStar software (DNAStar, Inc., Madison, WI). The primers were designed as previously reported [17-19]. Novel coding sequence variants detected in the patients were tested against the ethnically matched healthy controls. The alignment was also performed using UniProt (www.uniprot.org/uniprot/). In addition, variants identified in the study were also searched in the genome reference databases, including 1000Genomes (www.internationalgenome.org/) and Exome Aggregation Consortium (ExAC, http://exac.broadinstitute.org) databases. For sporadic EOAD cases, a group of six microsatellites markers, including D14S268, D14S620, D14S1028, D14S77, D14S1004, and D14S1025, was analyzed in the family to check parenthood.

### Bioinformatics analysis

Five different bioinformatics analyses based on evolutionary, structural, and computational methods were carried out to predict mutations *in silico*. Online bioinformatics programs were employed to evaluate the biological impact of the missense mutation *in silico*: sProtein Variation Effect Analyzer (PROVEAN, http://provean.jcvi.org/index.php) [20], MutPred (http://mutpred.mutdb.org/) [21], SNAP (www.rostlab.org/services/SNAP/) [22], PolyPhen2 (http://genetics.bwh.harvard.edu/pph2/) [23], Mutation Taster (www.mutationtaster.org) [24] and MutationAssessor (http://mutationassessor.org/) [25]. Homology modeling of the PSEN1 protein was also performed. The tertiary structure of the protein was downloaded from the SWISS-MODEL Repository (http://swissmodel.expasy.org/repository), and the effect of the mutations was evaluated in DeepView/Swiss-PdbViewer (version 4.0.4, http://spdbv.vitalit.ch/) [26].

**Table 1:** The clinical characteristics of the proband in the three EOAD families.

Clinical characteristics	Proband in family 1 (III-3)	Proband in family 2 (III-4)	Proband in family 3 (II -2)
Gender	Female	Female	Female
Family history	+	+	-
Age onset (years)	40	41	30
Disease process (years)	5	1	4
Symptoms onset	Memory decline	Memory decline	Memory decline
Psychiatric symptoms	+	+	+
Epilepsy	+	+	+
Extrapyramidal symptoms	+	-	-
MMSE	12	17	23
MoCA	10	-	19
CDR	2	1	1
ADAS-cog	NA	NA	23
Cranial MRI	Cerebral cortex and bilateral hippocampal atrophy	Bilateral frontoparietal lobe demyelination	Cerebral cortex and bilateral hippocampal atrophy

MMSE: Mini-Mental State Examination; MoCA: Montreal Cognitive Assessment); CDR: Clinical Dementia Rating; ADAS-cog: Alzheimer’s Disease Assessment Scale-cognitive; NA: not available

**Table 2:** Bioinformatic analyses of *PSEN1* variants.

Pedigrees	Family 1	Family 2	Family3
Nucleotide Change	c.766T>A	c.641A>G	c.641A>G
Amino Acid Change	p. Y256N	P. H214R	P. G206V
Location	Exon7, TM-VI	Exon7, HL-IV	Exon7, TM-IV
ExAC	0	0	0
1000Genome	0	0	0
PROVEAN Scores	-7.2 (Deleterious)	-7.6 (Deleterious)	-8.5 (Deleterious)
MutPred2 scores	0.953 (Pathogenicity)	0.934 (Pathogenicity)	0.926 (Pathogenicity)
SNAP scores	92 (functional effect)	85 ((functional effect)	75 (functional effect)
Polyphen 2 scores (HumDiv)	1.000 (Probably damaging)	1.000 (Probably damaging)	1.000 (Probably damaging)
Polyphen 2 scores (HumVar)	0.999 (Probably damaging)	0.999 (Probably damaging)	1.000 (Probably damaging)
MutationTaster	Disease causing	Disease causing	Disease causing
MutationAssessor	High deleterious	Medium deleterious	Medium deleterious

## RESULTS

### Clinical findings and genetic analysis

The clinical characteristics of probands in the three EOAD families are shown in Table 1. Sanger sequencing detected three mutations in exon 7 of the *PSEN1* gene in the three families. Two novel mutations of *PSEN1* were identified in families 1 and 2, respectively. Additionally, a *de novo* mutation was found in a vEOAD case in family 3.

#### Family 1 (*PSEN1* Y256N)

**Figure 1.**
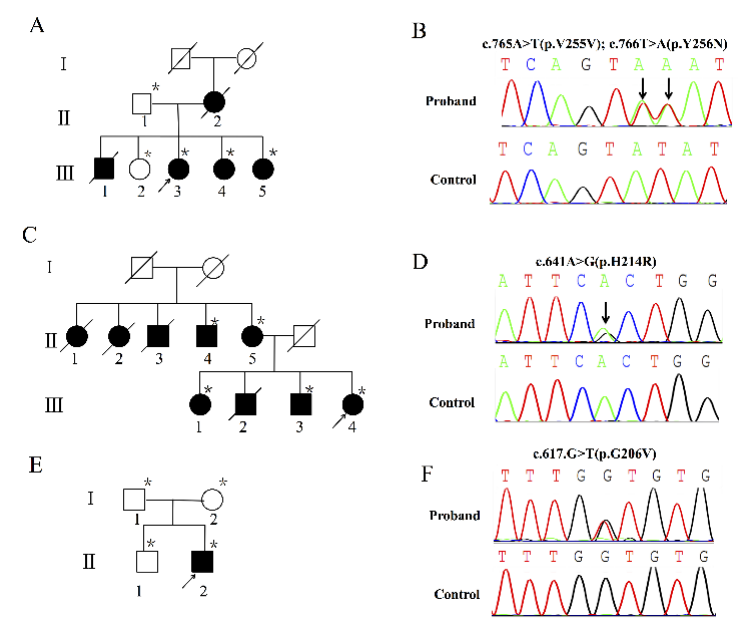
The pedigree charts and Sanger sequence chromatograms of the three families A) The pedigree chart of family 1. B) The Sanger sequence chromatogram of family 1. C) The pedigree chart of family 2. D) The Sanger sequence chromatogram of family 2. E) The pedigree chart of family 3. F) The Sanger sequence chromatogram of family 3. Open symbols, unaffected; filled symbols, affected; arrow, proband. Asterisk, members sequenced in the study. Vertical arrows indicate the mutation site.

**Figure 2.**
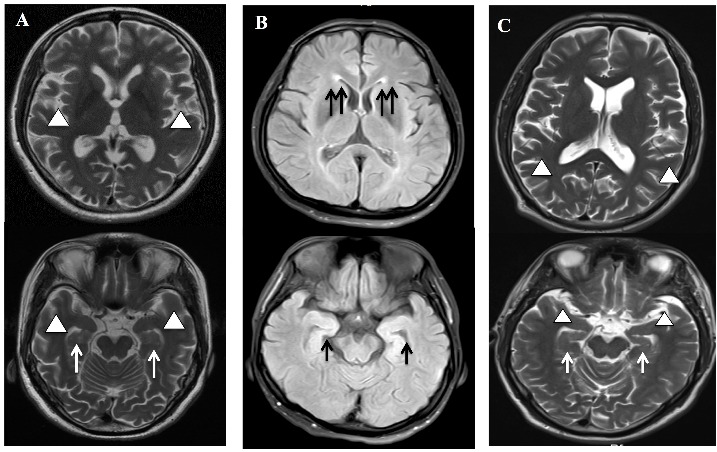
The cranial MRI of the probands in the three families A) The T2 weighed image showed atrophy of cerebral cortex (arrowhead) and hippocampus (arrow) in proband (III-1) in family 1. B) The T2 FLAIR image showed mild white matter demyelination (double arrows) in frontoparietal lobe of the proband (III-2) in family 2. C) The T2 weighed MRI image showed atrophy of cerebral cortex (arrowhead) and hippocampus (arrow) in proband (III-3) of family 3.

The proband (Fig. 1A, III-3) was a 45-year-old right-handed woman. She presented with slowness in response, short-term memory impairment at 40 years of age, and progressive deterioration combined with disorientation, confusion, and cognitive disorders (mental, calculative, and comprehensive disabilities). She got the diagnosis of EOAD at age 41 years (MMSE score = 12, MoCA score = 10, CDR score = 2). Cranial magnetic resonance imaging (MRI) uncovered bilateral temporal lobe and hippocampal atrophy (Fig. 2A). She has also had static tremor, kinetic tremor, hypermyotonia, speech disorder, and generalized tonic-clonic seizures for 42 years of age.

The proband’s mother (II-2) presented with a severe memory decline and died at 45 years of age. Her older brother (III-1) had a memory decline and mental disorders for 7 years and died at age 47 years of age. Her 39-year-old sister (III-4) and another 38-year-old sister (III-5) also had memory impairment for 3 and 5 years, respectively.

Genetic analysis revealed a synonymous mutation and an adjacent nonsynonymous mutation in the *PSEN1* gene in the proband, leading to a silent mutation, V255V, and a missense tyrosine-to-asparagine change, Y256N (Fig. 1B). Besides, other EOAD-affected family members (III-4, III-5) had the same mutation, but it was absent in unaffected relatives (II-1, III-2).

#### Family 2 (*PSEN1* H214R)

The proband (Fig. 1C, III-4) was a 42-year-old, right-handed woman. She presented with memory decline, and mood and behavioral disorders at 41 years of age. The MMSE score was 17, and the CDR score was 2. Cranial MRI showed mild white matter demyelination of the frontal and parietal lobes but no obvious cerebral cortex or hippocampal atrophy (Fig. 2B).

The family history revealed that proband’s three siblings (III-1, III-2 and III-3), her mother (II-5), and her mother’s siblings (II-1, II-2, II-3, and II-4) were also affected by the same symptoms at onset of age at ~40 years of age. Her older sister (III-1) and older brother (III-2) had the age onset of 40 years old and died 10 years later. Her 44-year-old brother (III-3) has had a slight memory decline for 41 years of age.

A heterozygous nucleotide substitution in exon 7 of the *PSEN1* gene, c.642A>G (p. His214Arg), was identified in the proband (III-4; Figure 1D). This mutation was also found in his mother, his older brother, and his uncle (II-5, III-3, and II-4).

#### Family 3 (*PSEN1* G206V)

The proband (Fig. 1E, II-2) was a 34-year-old right-handed male, who developed slowly progressing memory loss combined with irritation and anxiety at 30 years of age. His MMSE, MoCA, and CDR scores were 23, 19,and 1, respectively. The cranial MRI showed bilateral temporal lobe and hippocampal atrophy (Fig. 2C). His parents were nonconsanguineous, and none of his family members had similar symptoms.

DNA sequencing analysis revealed a heterozygous mutation (c.617G>T, p.Gly206Val) in *PSEN1* in the proband (Fig. 1F), but no additional AD-affected family member was found. DNA microsatellite markers analysis indicated that the mutation had occurred *de novo*.

### In silico predictions

**Figure 3.**
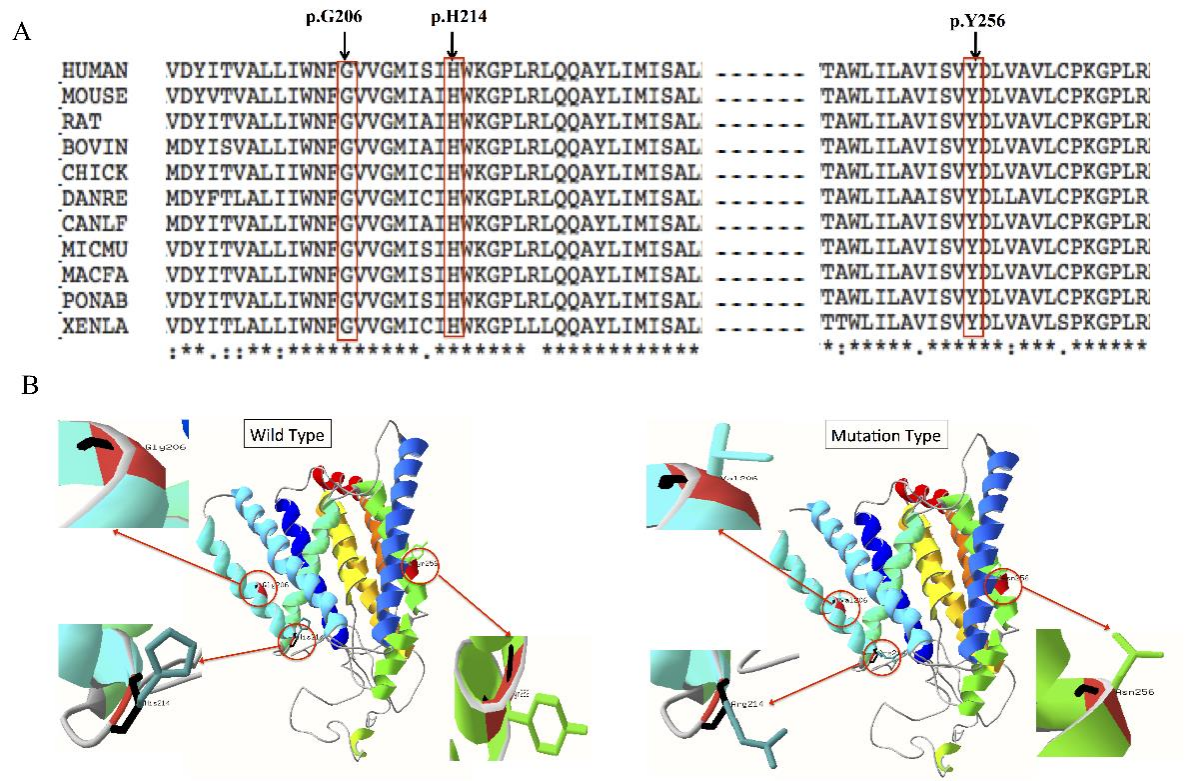
The conservation analysis of the three mutation sites and protein structure modeling of PSEN1 A) conservation analysis of the three mutation sites. Arrow indicates the mutation sites. B) the 3D protein structure modeling wild type and mutation type of PSEN1 protein. The residues in codons 206, 214, and 256 were highlighted. Mutations of Y256N, H214R, and G206V change the residue side chains at the positions 256, 214, and 206 of PSEN1 protein.

The bioinformatics analysis tools predicted that all the mutations were deleterious (Table 2). All the three mutated residues were found to be highly conserved across different species (Figure 3A). Furthermore, all the three mutations were absent in the 200 ethnically matched healthy controls and have not been reported in the dbSNP database or 1000 Genomes Project database. The tertiary structure of protein PSEN1 with wild-type residues or mutations was generated. Mutations Y256N, H214R, and G206V resulted in changes at positions 256, 214, and 206 of the amino acid side chains of the PSEN1 protein (Fig. 3B).

## DISCUSSION

In this study, we analyzed three Chinese EOAD families. Three mutations in exon 7 of the *PSEN1* gene were identified, including two novel mutations (Y256N and H214R) in two EOAD families, and a *de novo* mutation (G206V) in an unrelated family in a patient with vEOAD. Exon 7 of *PSEN1* is one of the most frequently mutated loci, and mutations in this exon have often been found in association with vEOAD [27].

A novel mutation of* PSEN1* (Y256N) was identified in this study, which is a second case with a mutation at this position. Previously, Y256S in *PSEN1* has been reported to be associated with one of the youngest ages of AD onset (25 years old). The patient presented with aggressive vEOAD with severe involvement of the primary motor cortex [28]. In contrast, the vEOAD-affected patients with the Y256N mutation in our study had milder clinical symptoms than did the patients with the Y256S mutation, and a relative older age at onset (40 years old). The Y256N mutation may be less harmful than the previously reported Y256S mutation, and more studies are needed to further test this hypothesis.

The second novel mutation C.641A>G (H214R) in *PSEN1* was identified in family 2. Two known mutations have been reported at this amino acid positon. The H214Y mutation was detected in two families with the age of onset ranging from 37 to 51 years old, with the varied clinical phenotypes in the same family [29, 30]. Another mutation, H214D, was also detected in one EOAD family, in which the proband developed AD symptoms at the age of 55 years [31]. Although the underlying pathogenesis remains unclear, the H214R mutation may not be as harmful as other mutations that caused earlier onset of AD and typical cerebral cortex atrophy.

A *de novo* mutation in *PSEN1*, G206V, was identified in a vEOAD case in family 3. The mutation has been reported previously in one autosomal dominant EOAD family with four affected patients in three generations. They developed memory loss in their late 20s to early 30s and died in their early 40s [32]. This is the second report on this mutation to date, and we proved the previously published hypothesis that G206V in *PSEN1* is a probable pathogenic mutation. Besides, other mutations (G206S, G206A, and G206D) at this amino acid position have been also reported, suggesting a mutation hotspot at this locus [33-35]. One of the largest cohort studies has identified nine *de novo* mutations in the *PSEN1* gene in 129 sporadic cases with onset below age 51 [9]. This evidence also suggests that genetic analysis should be performed not only in familial EOAD, but also in sporadic EOAD.

Emerging research projects in Japan, Korea, and China are evaluating the genotype and clinical phenotype of AD [14]. According to the Alzheimer’s Research Forum database (www.alzgene.org), 253 variants have been discovered in *PSEN1* around the world. Among them, 58 variants have been discovered in Japan, Korea, China, or Malaysia. Because the aging population in most Asian countries is increasing, determining the initial pathological change is a key issue for AD research and treatment. Genetic analysis helps with AD diagnosis, evaluation of the pathogenesis, and finding suitable therapeutic targets in AD [15].

In conclusion, we reported two novel mutations in *PSEN1* (Y256N and H214R) and a de novo mutation (*PSEN1* G206V) detected in three Chinese EOAD families. Nevertheless, further functional studies on the three mutations are needed to determine their roles in the pathogenesis of AD.
